# Understanding Actinomyces Odontolyticus: A Rare Culprit of Bacteremia

**DOI:** 10.7759/cureus.66086

**Published:** 2024-08-03

**Authors:** Sujeirys Paulino, Maria Duran, Nishant Allena, Franklin Sosa, Ravish Singhal

**Affiliations:** 1 Internal Medicine, BronxCare Health System, Bronx, USA; 2 Medicine, BronxCare Health System, Bronx, USA; 3 Pulmonary Medicine, BronxCare Health System, Bronx, USA; 4 Pulmonary and Critical Care Medicine, BronxCare Health System, Bronx, USA

**Keywords:** actinomyces odontolyticus bacteremia, a. odontolyticus, actinomyces odontoliticus, actinomyces, actinomyces bacteremia

## Abstract

*Actinomyces *species are gram-positive filamentous non-acid fast anaerobic to microaerophilic bacteria that belong to human oral, gastrointestinal, and urogenital tract flora. Cervicofacial, abdominopelvic, and pulmonary infections are the most common presentations. Hematogenous spread is extremely rare and has been associated with *Actinomyces meyeri, Actinomyces israelii, and Actinomyces odontolyticus*. It affects individuals with poor oral hygiene, heavy alcohol intake, immunosuppressed, and underlying pulmonary diseases typically between the second and sixth decades of life with the peak incidence being between the fourth and fifth decades. We present a case of *A. odontolyticus* bacteremia in a patient with uncontrolled diabetes mellitus and chronic sinusitis.

## Introduction

Actinomycosis is an uncommon infection characterized by granulomatous and suppurative inflammation, primarily caused by anaerobic gram-positive *Actinomyces*. Data on the exact prevalence of actinomycosis is scarce, but reports suggest a significant decline in its incidence over recent decades. Disseminated actinomycosis, which can spread to various body parts, occurs in roughly 20% of cases, with the central nervous system being affected in 1%-2% of those instances. Approximately 70% of infections are due to either *Actinomyces israelii* or *Actinomyces gerencseriae* [1-3].

As a member of the *Actinomyces *genus, *Actinomyces odontolyticus* is rarely encountered in clinical practice, yet it remains an intriguing culprit of different types of infections, mainly dental, oropharyngeal, and abdominopelvic [1,3]. Bacteremia caused by this bacterium is extremely rare, and case reports are sparse. The identification of *Actinomyces odontolyticus* remains a diagnostic challenge due to the need for specialized microbiology techniques and the fastidious nature of the organism [3]. Medical management of *A. odontolyticus *bacteremia encompasses a multidisciplinary approach consisting of antibiotics tailored to the organism’s susceptibility, source control from the primary focus of infection, and management addressed to treat the septic complications along with supportive care [4-7].

With this case report, we aim to strengthen the understanding of *A. odontolyticus* and highlight the importance of this rare pathogen as a possible cause of bacteremia and increase awareness of the impact of this rare yet clinically significant bacteria due to its pathogenic potential.

## Case presentation

We present a case of a 65-year-old female with a medical history significant for moderate persistent bronchial asthma with multiple episodes of exacerbation requiring endotracheal intubation and mechanical ventilation, uncontrolled diabetes mellitus, hypertension, and ischemic cerebrovascular disease with residual right-sided weakness, who presented for worsening shortness of breath, persistent wheezing, and nonproductive cough for three days.

Upon arrival at the hospital, she was tachycardic (110 beats per minute (bpm)) and tachypneic (21 bpm), with an oxygen saturation of 99% on room air. Physical examination was consistent with bilateral expiratory wheezing; however, she appeared comfortable and was able to speak in complete sentences. Respiratory culture was positive for influenza B virus. Her initial blood work was unremarkable, except for respiratory and lactic acidosis (Table 1).

**Table 1:** Arterial blood gas

Parameters	Results	Reference range
pH	7.27	7.35-7.45
PCo_2_	63.4 mmHg	35-45 mmHg
Po_2_	65.3 mmHg	83-108 mmHg
Bicarbonate	28 mmoles/L	22-28 mmoles/L
Lactic acid	2.5 mmoles/L	0.5-1.1.6 mmoles/L

**Figure 1:**
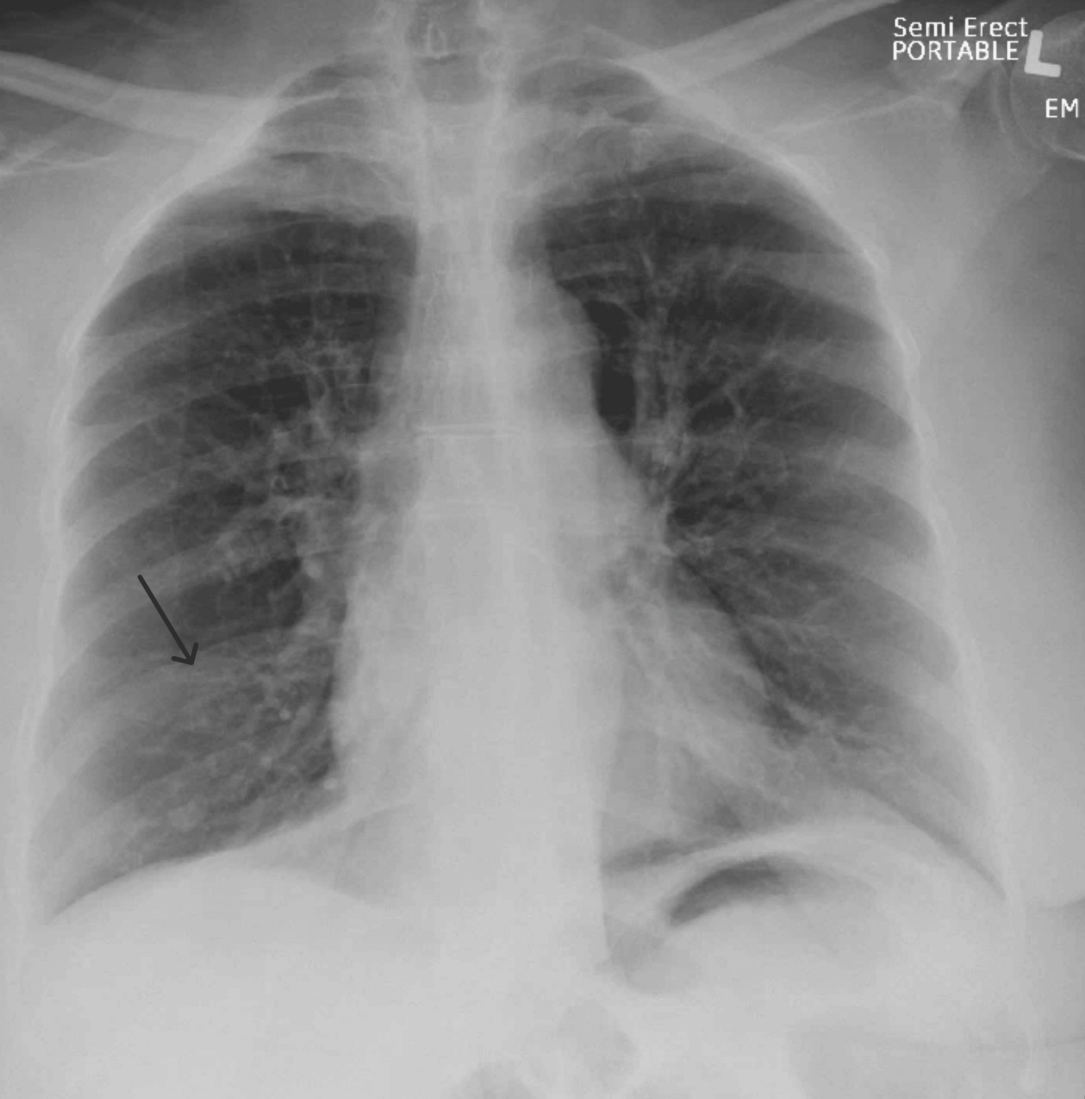
AP chest X-ray Mild bilateral peribronchial cuffing and prominent left lower lobe peribronchial markings were observed. These findings were suggestive of a small airway process without a dense focal infiltrate to suggest bacterial pneumonia (marked by a black arrow). AP: Anterior-posterior.

X-ray of the chest taken on admission was suggestive of a small airway process (Figure 1). She was started on treatment for asthma exacerbation with steroids, bronchodilators, and nebulizer treatments. In addition, she was started on oseltamivir for influenza.

Later, on admission day, the patient's clinical status worsened, she developed respiratory distress, became tachycardic with a heart rate of 125 bpm, and tachypneic to 35 respirations per minute. She was unable to speak in full sentences and was lethargic, which prompted the medical team to perform endotracheal intubation with mechanical ventilation. Respiratory and blood cultures were collected; the first culture was negative for pathogenic bacteria, but the second culture showed the presence of *A. odontolyticus*. Infectious disease was consulted, and clindamycin 600 mg every six hours was added to the regimen as our patient was allergic to penicillin.

**Figure 2:**
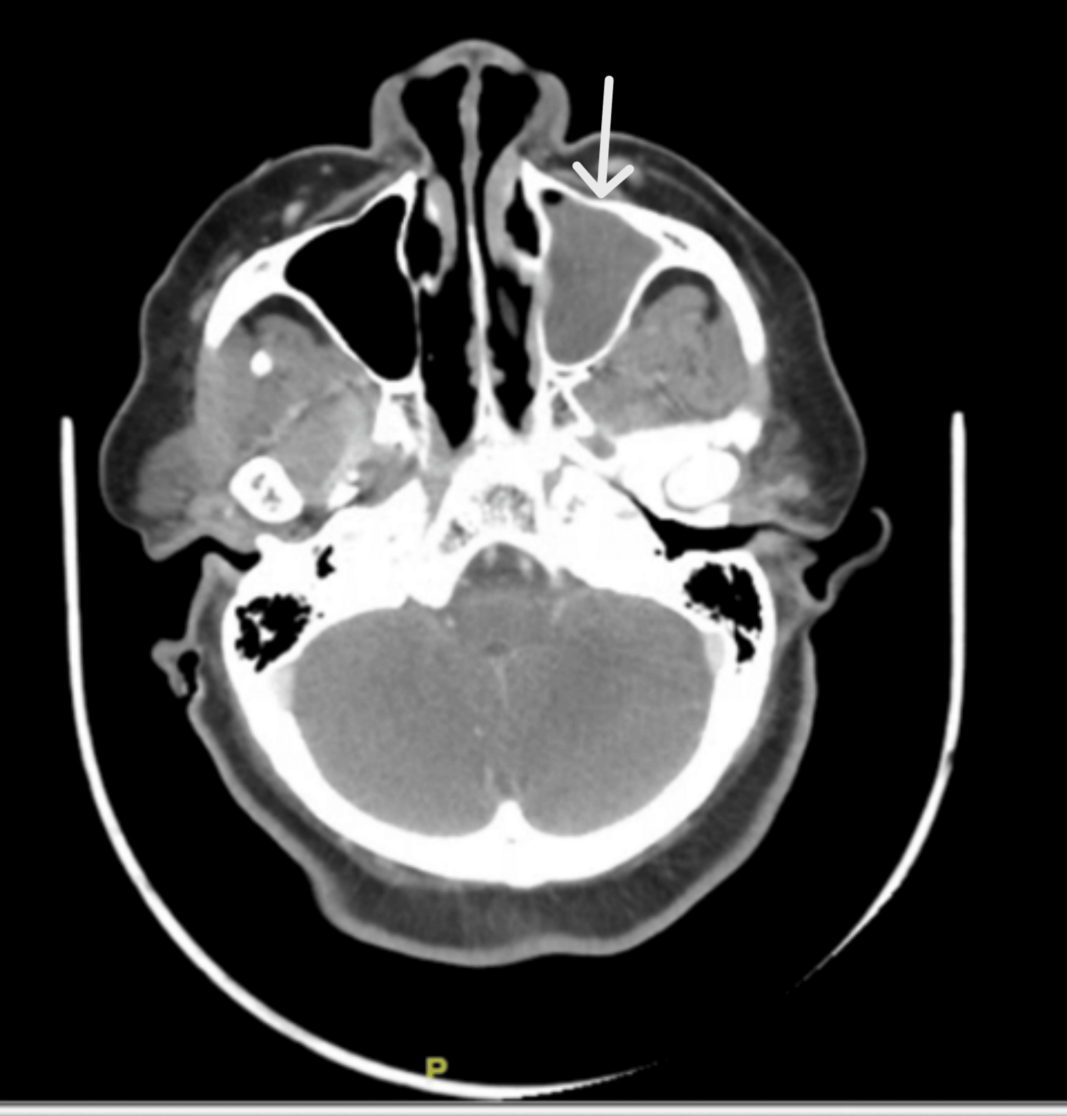
The axial section of the head CT showing opacification of the left maxillary sinus (marked by the white arrow)

**Figure 3:**
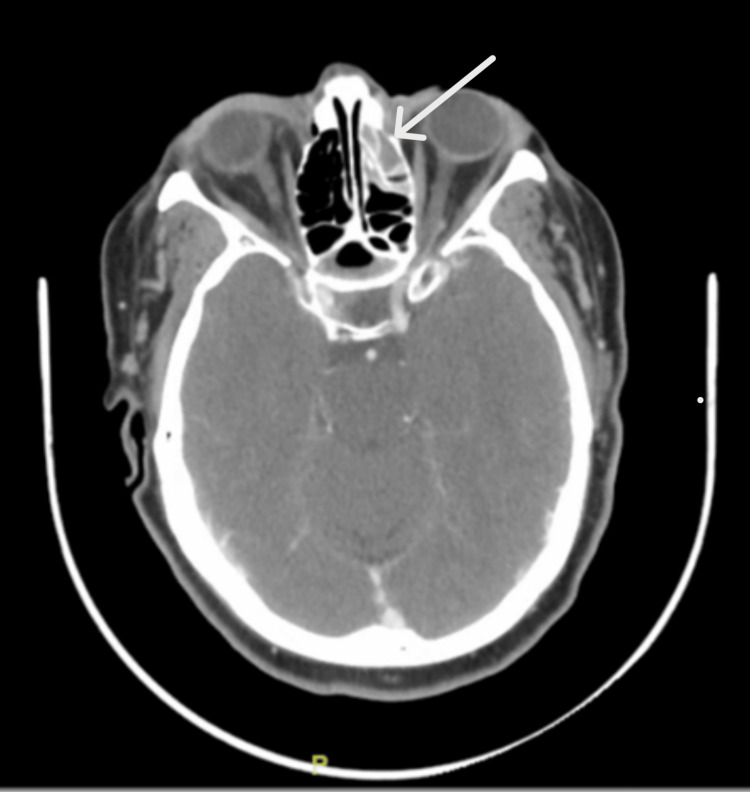
The axial section of the head CT showing opacification of the inferior aspect of the left frontal sinus and left sphenoid sinus (marked by the white arrow)

Various computerized tomography scans (CT scans) with contrast were performed to look for the source of the infection. CT of the chest, abdomen, and pelvis did not reveal any thoracic or abdominopelvic source. However, CT of the head and neck reported opacification of the inferior aspect of the left frontal sinus, left ethmoid air cells, sphenoid sinuses, and left maxillary sinus with air-fluid levels, representing sinusitis (Figures 2, 3).

On the fifth day of admission, she was liberated from mechanical ventilation and reported a history of frequent headaches and occasional nasal congestion for the past three months. She was treated with clindamycin for 10 days, with a resolution of the symptoms. On post-hospitalization follow-up, she felt well and had no symptoms.

## Discussion

*Actinomyces *species are gram-positive filamentous non-acid fast anaerobic to microaerophilic bacteria that belong to human oral, gastrointestinal, and urogenital tract flora. Infections of the skin, bone, joints, respiratory, and genitourinary tract are described and frequently associated with abscess and sinus tract formation, but bacteremia happens rarely. *Actinomyces *species are genetically related to *Mycobacterium *and *Nocardia *species. Therefore, active infection, although rare, can mimic tuberculosis, nocardiosis, or malignancy presenting a diagnostic challenge to physicians [1,3,8].

This disease is more common during the second and sixth decades of life, with the peak incidence between the fourth and fifth decades. Males are affected more than females, with a ratio of 3:1, especially in patients with low socioeconomic status [3]. Frequently affected individuals are those with poor oral hygiene, heavy alcohol users, and those with underlying pulmonary diseases such as chronic bronchitis, emphysema, bronchiectasis, and a history of pulmonary tuberculosis [1].

Hematogenous spread is extremely rare and has been associated with *A. meyeri*, *A. israelii*, and *A. odontolyticus* [1]. In 2022, Ali et al. published a five-year retrospective study on *A. Odontolyticus* infection, in which they emphasized the rarity of this presentation. They stated that in the four decades following the isolation of this bacteria, around 20 cases of bacteremia have been identified in different countries, including the United States [5]. Risk factors that predispose patients to develop bacteremia by *Actinomyces *are not completely understood; however, chronic sinusitis was a risk factor identified in two of the cases with bacteremia, similar to our patient in this case report. Diabetes mellitus is another frequent comorbidity, especially in adults who develop bacteremia [1,4].

The pathophysiology of invasive disease after oral mucosa lesion is not well known, but the presence of other oral commensals is considered to play a role in inhibiting local host defenses and decreasing the oxygen tension that facilitates *Actinomyces *inoculation. Cervicofacial, abdominopelvic, and pulmonary infections are the most common types of infections [1,3-5]. The presence of immunosuppression has been shown to play a role in the development of bacteremia, as reported in patients with renal and lung transplants, those with human immunodeficiency virus (HIV), steroid and immunosuppressant users, patients with cancer, and intravenous drug users [1,6,7].

To date, there are very few cases of patients with uncontrolled diabetes who develop *Actinomyces *infection. It could be hypothesized that through diabetes-induced immune dysfunction, alterations in oral microbiota, periodontal disease, vascular complications, and reduced tissue oxygenation, patients with uncontrolled diabetes are at higher risk for this type of infection compared to the general population [1].

The identification of bacteria remains elusive as culture from infected tissue yields no growth in more than 50% of cases. Usually, Gram staining is more sensitive for identification. Granuloma formation is common, and sulfur granules are found in 75% of cases. Regarding drug susceptibility, *Actinomyces *species are susceptible to beta-lactam antibiotics, with penicillin G or amoxicillin being the most commonly used. In case of allergy, doxycycline, clindamycin, and macrolides have been used [1,9].

Although *A. odontolyticus* bacteremia is rare, it does not always require treatment as demonstrated by a study series done in the United Kingdom in which 60 patients with positive blood culture for *A. odontolyticus* were identified. Out of those, only 10 received treatment based on the presence of active actinomycoses, defined as pulmonary actinomycosis, abdominal, dental, multiple sites, soft tissue, and not categorized. The rest of the patients who did not receive antibiotic therapy had no apparent negative clinical outcomes, and no further hospitalizations could be attributed to this bacteremia [10].

## Conclusions

The identification of *A. odontolyticus* as a cause of bacteremia is an infrequent event, with only a few cases being reported. Based on previous reports, the treatment is based on clinical presentation and medical judgment, but more studies need to be conducted to determine the treatment guidelines for this entity.
